# Direct-Acting Oral Anticoagulants: Practical Considerations for Emergency Medicine Physicians

**DOI:** 10.1155/2016/1781684

**Published:** 2016-05-16

**Authors:** W. Frank Peacock, Zubaid Rafique, Adam J. Singer

**Affiliations:** ^1^Section of Emergency Medicine, Baylor College of Medicine, Ben Taub General Hospital, 1504 Taub Loop, Houston, TX 77030, USA; ^2^Department of Emergency Medicine, Stony Brook School of Medicine, University Medical Center L4, 100 Nicolls Road, Stony Brook, NY 11794-8350, USA

## Abstract

Nonvalvular atrial fibrillation- (NVAF-) related stroke and venous thromboembolism (VTE) are cardiovascular diseases associated with significant morbidity and economic burden. The historical standard treatment of VTE has been the administration of parenteral heparinoid until oral warfarin therapy attains a therapeutic international normalized ratio. Warfarin has been the most common medication for stroke prevention in NVAF. Warfarin use is complicated by a narrow therapeutic window, unpredictable dose response, numerous food and drug interactions, and requirements for frequent monitoring. To overcome these disadvantages, direct-acting oral anticoagulants (DOACs)—dabigatran, rivaroxaban, apixaban, and edoxaban—have been developed for the prevention of stroke or systemic embolic events (SEE) in patients with NVAF and for the treatment of VTE. Advantages of DOACs include predictable pharmacokinetics, few drug-drug interactions, and low monitoring requirements. In clinical studies, DOACs are noninferior to warfarin for the prevention of NVAF-related stroke and the treatment and prevention of VTE as well as postoperative knee and hip surgery VTE prophylaxis, with decreased bleeding risks. This review addresses the practical considerations for the emergency physician in DOAC use, including dosing recommendations, laboratory monitoring, anticoagulation reversal, and cost-effectiveness. The challenges of DOACs, such as the lack of specific laboratory measurements and antidotes, are also discussed.

## 1. Introduction

Stroke is associated with nonvalvular atrial fibrillation (NVAF), occurring in a yearly average of 5% of untreated NVAF patients and equaling approximately 700,000 cases per year [1]. Venous thromboembolism (VTE), including deep-vein thrombosis (DVT) and pulmonary embolism (PE), occurs at a rate of 117 people per 100,000 person-years annually, with increased incidence in select patient populations [2, 3]. Both NVAF-related stroke and VTE are causes of significant economic burden. In the United States, the healthcare costs associated with VTE exceed $1.5 billion per year, and the direct cost to treat the first year of AF-related strokes is $2.6 billion (2003 US dollars) [1, 4, 5].

The historical standard of care for VTE treatment is initiated by the administration of a parenteral anticoagulant for 5 to 10 days, followed by overlapping treatment with the vitamin K antagonist (VKA) warfarin [6]. The parenteral anticoagulant options for VTE treatment include intravenous unfractionated heparin, subcutaneous low-molecular-weight heparin (LMWH) (e.g., enoxaparin), or fondaparinux [6]. Intravenous administration requires vascular access, and subcutaneous injections may lead to increased patient discomfort and injection hematoma [7]. Patients receiving heparin are at risk for heparin-induced thrombocytopenia, although this risk is lower for LMWH than for unfractionated heparin [8, 9]. Despite the convenience of oral administration, the use of warfarin is complicated by delayed onset of action, narrow target therapeutic range, unpredictable dose responses, and numerous food and drug interactions [10, 11]. Patients taking warfarin also require frequent monitoring, as variable levels of anticoagulation increase the risk for both recurrent thromboembolism and bleeding [10, 11]. In a study of 395 patients receiving warfarin in a US Veterans Affairs facility, the estimated annual total cost of preventable warfarin-related adverse events (AEs) was $270,000 [12].

Dabigatran (Pradaxa®; Boehringer Ingelheim Pharmaceuticals, Inc., Ridgefield, CT), rivaroxaban (Xarelto®; Janssen Pharmaceuticals, Inc., Titusville, NJ), apixaban (Eliquis®; Bristol-Myers Squibb Co., Princeton, NJ), and edoxaban (Savaysa® (United States) and Lixiana® (European Union and Japan); Daiichi Sankyo, Parsippany, NJ) are direct-acting oral anticoagulants (DOACs) that have been approved in many regions of the world for the prevention of stroke or systemic embolic events (SEE) in patients with NVAF and for the treatment of VTE [13–18]. Direct-acting oral anticoagulants act by inhibiting a single component in the coagulation cascade: either factor Xa (rivaroxaban, apixaban, and edoxaban) or thrombin (dabigatran) [13–16]. Compared with warfarin, the advantages of DOACs include much faster onset of action, simpler dosing, reduced monitoring requirements, reduced food and drug interactions, and a decreased risk of bleeding [13–16]. The properties of DOACs are summarized in Table 1.

## 2. Summary of Efficacy and Safety of DOACs in Phase 3 Clinical Trials

In the pivotal phase 3 clinical trials in patients with NVAF, the 4 DOACs were at least as effective as warfarin in reducing the risk of stroke or SEE (see Supplementary Table  1 in Supplementary Material available online at http://dx.doi.org/10.1155/2016/1781684). In addition, DOACs were associated with similar or decreased rates of major bleeding, and patients experienced decreased rates of intracranial bleeding compared with warfarin [19–22]. A meta-analysis of these trials confirmed that DOACs were associated with reduced risk of stroke or SEE as well as decreased rates of all-cause mortality and intracranial hemorrhage (ICH) compared with warfarin [23]. However, nonfatal gastrointestinal (GI) bleeding events were increased with the use of DOACs relative to warfarin [23]. While the exact reasons for decreased ICH remain unclear, hypotheses include that DOACs target a single component (rather than multiple components) in the coagulation cascade and have no direct effect on factor VIIa, a vitamin K-dependent coagulation factor whose receptor (tissue factor) is expressed in high levels in the blood vessels of the brain [24–26]. The increased GI bleeding associated with DOACs may be attributed to the lower absorption of DOACs across the GI mucosa relative to that of warfarin [27]. With incomplete DOAC absorption, the active anticoagulant in the GI tract lumen may aggravate bleeding from vulnerable lesions [27].  Table 2 summarizes the bleeding outcomes of DOACs from phase 3 clinical trials for the prevention of stroke and SEE in patients with NVAF.

In the pivotal phase 3 clinical trials in patients with VTE, DOACs were noninferior for the treatment of acute symptomatic VTE and were associated with significantly decreased bleeding risks compared with standard therapy (LMWH or unfractionated heparin followed by treatment with an overlapping VKA (warfarin or acenocoumarol); see Supplemental Table  2) [29–33].  Table 3 summarizes the bleeding outcomes of DOACs from phase 3 clinical trials for the treatment and secondary prevention of VTE.

## 3. DOACs for the Treatment of DVT or PE

Pulmonary embolism is a potentially life-threatening condition that, in the US, has historically resulted in hospitalization in a vast majority of cases. This compares unfavorably with DVT, as 1 cost-effectiveness study has shown that approximately 40% of DVT patients are treated entirely in an outpatient fashion [39]. Among patients with DVT, the need for hospitalization is determined by the presence of any of the following criteria: massive DVT, high risk for anticoagulant-related bleeding, uncontrolled pain, signs of limb ischemia, or another comorbidity that warrants in-hospital care [40]. Before the approvals of DOACs, most DVT patients received initial therapy with an LMWH (e.g., enoxaparin) as a lead-in combined with warfarin as primary treatment; this allowed for the opportunity to educate patients on heparin administration and make arrangements for follow-up visits for warfarin monitoring.

Like other oral anticoagulants, DOACs can increase the risk for bleeding [41]. Therefore, bleeding risk assessment is important for the determination of anticoagulant regimens. Limited evidence suggests that several DOACs and warfarin may have similar bleeding risk factors [42]. In general, clinical factors that may increase a patient's warfarin-associated bleeding risk include advanced age; hypertension; malignancy; bleeding history; certain concomitant medications; and comorbidities such as heart failure, diabetes, and renal or hepatic diseases [43]. Furthermore, most bleeding risk factors, except for concomitant medications, are also risk factors for adverse VTE outcomes. This requires physicians to carefully balance the risks for an adverse VTE event, such as VTE recurrence [44], chronic thromboembolic pulmonary hypertension [45], and death [44], against the risk of bleeding when using anticoagulants. Notably, DOACs increase the risk for GI bleeding (which is rarely fatal) but decrease the risk for ICH (which is commonly fatal) compared with warfarin. Thus, a challenge of using DOACs is to manage their bleeding risk profile, which differs from that of warfarin, to minimize the risk of stroke or an adverse VTE event [46]. Given the complexity of assessing bleeding potential, tools have been developed to quantify the bleeding risk for an individual taking oral anticoagulants [43]. For example, the HAS-BLED scoring tool evaluates common bleeding risk factors such as hypertension, abnormal renal/liver function, stroke, and bleeding history [47].

For the treatment of DVT or PE, DOAC dose regimens vary between the individual agents and may be influenced by patient characteristics. For example, dabigatran and edoxaban require heparinoid lead-in, while apixaban and rivaroxaban start with higher initial doses that are subsequently lowered to a maintenance dose [13–16]. It should be noted that initiation of rivaroxaban or apixaban is not recommended as an alternative to unfractionated heparin in patients with PE who present with hemodynamic instability or who may receive thrombolysis or pulmonary embolectomy [13, 15]. Further, most DOACs either require dose adjustment or are not recommended for patients with renal insufficiency [13–16]. Finally, while DOACs have fewer drug-drug interactions than warfarin, they do carry some concomitant drug restrictions. All DOACs are substrates of P-glycoprotein (P-gp), a cell membrane efflux transport protein that is known to pump drugs out of cells [48] and are metabolized by cytochrome P450 3A4 (CYP3A4), a family of oxidases that metabolizes drugs, to varying degrees, with dabigatran and edoxaban having the lowest metabolic dependence on CYP3A4 [49]. Thus, concomitant use of P-gp or CYP3A4 modulators may alter DOAC exposure [13–16]. P-gp or CYP3A4 inducers decrease DOAC exposure and thereby increase the risk of VTE, while P-gp or CYP3A4 inhibitors generally increase DOAC exposure and may lead to increased bleeding risk. However, the effect of drug-drug interactions on DOAC exposure varies across the DOACs. A detailed list of DOAC dosing for treatment and secondary prevention of VTE is available in Table 4.

Patients who have undergone anticoagulation for the prevention or treatment of VTE are at an increased risk for VTE recurrence after the discontinuation of anticoagulants [50]. However, the risk of VTE recurrence upon anticoagulant cessation is not significantly higher than the initial risk of developing a VTE (should the patient not be anticoagulated), indicating no rebound effect for anticoagulant treatment. Data from phase 3 clinical trials indicate that patients treated with DOACs and standard therapy (heparin/VKA) have similar VTE recurrence risks as shown in Supplementary Table  2.

## 4. Assessing the Anticoagulant Effect of DOACs at the Time of Bleeding

Laboratory measurement of DOACs, though not required for regular use, may be necessary in circumstances of bleeding management or perioperative monitoring. While US Food and Drug Administration- (FDA-) approved measurements for rivaroxaban, apixaban, and edoxaban are unavailable, the anticoagulant activity of dabigatran can be detected using activated partial thromboplastin time (aPTT) [13–16, 51]. However, limitations still exist in the use of aPTT for dabigatran monitoring, including the lack of a defined therapeutic range [52, 53], low sensitivity to different plasma concentrations [52–54], and inapplicability in patients with lupus anticoagulant or intrinsic clotting factor deficiency that may prolong aPTT [55]. The ecarin clotting time (ECT) assay responds to dabigatran in a dose-dependent fashion and therefore can be used to quantify dabigatran concentrations [56, 57]. However, this approach is unavailable in real time for most hospital labs [58, 59]. Another method for dabigatran quantification is the thrombin time (TT) test, which can detect dabigatran with exquisite sensitivity. While the traditional TT cannot be used for quantification purposes because of the lack of standardization, commercially available assay kits based on calibrated dilute TT (dTT) can provide results linearly correlated with dabigatran plasma concentrations spanning the therapeutic range and, therefore, represent a rapid method for measuring dabigatran plasma levels [60].

Although rivaroxaban and apixaban affect both aPTT and PT, some studies suggest that PT, but not aPTT, may represent a relatively sensitive assessment for the activity of these 2 DOACs [52, 59, 61]. However, the utility of absolute PT for estimating anticoagulant activity varies greatly based on the specific assay used and timing relative to the last dose. Edoxaban may be measured by both aPTT and PT, but there is considerable variability among different reagents for each of these assays [62]. Thrombin generation-endogenous thrombin potential (ETP) has been put forth as a reliable measure of the anticoagulant effect of DOACs in multiple studies [63–66]. While commercial assay kits are available for thrombin generation-ETP, using this method for DOAC measurement requires standardization and primarily occurs in research laboratories rather than clinical practice. The chromogenic antifactor Xa assay may be used for quantitative assessment of oral FXa inhibitors. Although this method requires standardization, the availability of commercial assay kits along with DOAC-specific calibrators has enabled reliable DOAC quantification using antifactor Xa assay [52, 57, 60, 67–70].

Clinical guidance regarding bleeding management based on DOAC laboratory measurement is limited, but the recently published guidelines from the International Society on Thrombosis and Haemostasis (ISTH) have provided some insights [71]. Qualitative measurement of DOACs can help determine their contribution to bleeding or the timing for an unplanned procedure [71]. For example, as aPTT and TT are sensitive tests for dabigatran, a normal aPTT or TT result may exclude the actual effect of dabigatran in a patient who has received this agent [71]. In addition, quantification of DOAC levels contributes to the decision as to whether a reversal agent should be administered. Except for patients who experience life-threatening bleeding or require emergency surgery for life-threatening conditions, factors that influence the need for a reversal agent include the time of the last DOAC dose, creatinine clearance (CrCl), and the DOAC plasma concentration obtained from laboratory tests. In patients with serious bleeding, a drug concentration over 50 ng/mL may warrant antidote administration, while in patients requiring an urgent procedure associated with a high risk of bleeding, antidote use should be considered at a DOAC concentration exceeding 30 ng/mL [71]. Although pharmacokinetic studies have provided reference plasma concentration ranges for DOACs, the clinical significance of an out-of-range measurement remains unclear [60]. Also, patients' DOAC plasma concentrations and anticoagulant effect may vary over time [60]. Therefore, clinicians should interpret DOAC laboratory measurement results with caution and should not overreact at laboratory measurement results slightly beyond the reference range [60].

## 5. Management of Bleeding

### 5.1. General Approaches for Managing Oral Anticoagulant-Related Bleeding

Bleeding remains a serious risk for all patients receiving anticoagulants. Therefore, bleeding management is an important consideration in anticoagulation therapies. General approaches for managing DOAC- and warfarin-related bleeding are similar and include applying mechanical pressure or performing endoscopic/surgical procedures at the bleeding site, as well as using supportive measures [72]. Bleeding related to antithrombotic therapy can also be controlled by hemostatic agents or anticoagulant antidotes; however, these agents may differ between DOACs and warfarin.

### 5.2. Management of Warfarin-Related Bleeding

For managing bleeding in patients receiving warfarin, guidelines published by the American College of Chest Physicians (ACCP) recommend the use of vitamin K, fresh frozen plasma (FFP), prothrombin complex concentrate (PCC), or recombinant activated factor VIIa (rVIIa), depending on the urgency of the situation [10]. Because vitamin K is slow acting, concomitant administration of FFP or PCC may be necessary to speed the reversal process [73, 74]. However, the use of FFP is associated with significant limitations, including thaw time, the need for ABO blood type matching, potential pathogen transmission, and insufficient reversal due to varying levels of coagulation factors [75–77]. More importantly, FFP must be given in large volumes for patients with greatly elevated international normalized ratios (INRs), which may lead to the development of transfusion-associated circulatory overload (TACO), a type of noncardiogenic lung edema [78]. FFP may also induce transfusion-related acute lung injury (TRALI), another type of lung edema resulting from immunologic reactions to the transfusion components, although this occurs at a much lower rate than TACO [77, 78]. In contrast, PCC is easy to use and can be administered more quickly, with significantly decreased risks of pathogen transmission, TACO, and TRALI [74, 77, 79, 80]. For these reasons, PCC is recommended over FFP for the reversal of anticoagulation in clinical practice in patients with life-threatening bleeding. Although not specifically included in the guidelines, Kcentra® (CSL Behring LLC, King of Prussia, PA), a 4-factor PCC (clotting factors II, VII, IX, and X) (4F-PCC) that also contains protein S, protein C, and antithrombin, is approved by the FDA specifically for warfarin reversal in cases of acute major bleeding or in need of urgent surgery [81, 82]. In a pivotal phase 3 clinical trial, Kcentra was noninferior in achieving effective hemostasis and superior in reducing INR compared with FFP; the safety profile is similar between Kcentra and FFP [82].

### 5.3. Management of DOAC-Related Bleeding

Reversal agents for DOACs are very limited, and there are no reversal agents currently approved by the FDA specifically to reverse the anticoagulant effects of rivaroxaban, apixaban, or edoxaban. This poses a potential challenge to bleeding management or perioperative interruption, especially for emergency surgeries in patients receiving DOACs. However, DOACs may be associated with better outcomes after major bleeding than warfarin. A study comparing outcomes of major bleeding between patients receiving dabigatran and warfarin revealed that dabigatran-treated patients had a lower 30-day mortality rate, significantly shorter stays in the intensive care unit, and reduced need for plasma transfusions after major bleeding than those treated with warfarin, although the dabigatran-treated patients in the study were older, had lower creatinine clearance, and more frequently used aspirin or nonsteroidal anti-inflammatory agents [83]. In the pivotal clinical trials, rivaroxaban and apixaban were associated with lower rates of vitamin K-related medication uses and FFP transfusions after major bleeding than warfarin; patients receiving rivaroxaban who experienced major bleeding also had fewer PCC administrations and shorter hospitalization [84, 85]. A meta-analysis including studies of all 4 DOACs has also reported lower risks of death after major bleeding for DOACs than for warfarin [86]. In addition, DOACs are fast acting with relatively short half-lives, which may translate to a short periprocedural interruption in anticoagulation [13–16]. Practice guidelines suggest that heparin bridging for periprocedural DOAC interruption may not be required, as is the case for warfarin [87]. Circumventing the need for bridging not only improves convenience but also decreases the risk for major bleeding, especially in patients who undergo major procedures [88]. However, definitive guidance regarding periprocedural DOAC interruption is lacking because of limited clinical evidence and the rapid progression of this research field [89, 90]. In general, surgical procedures should be delayed for at least 24 hours after the discontinuation of DOACs, as indicated by current practice guidelines [87]. The effect of renal function, age, drug affected half-life, concomitant medications, and timing of the last dosage should be considered. The DOAC package inserts and some clinical guidelines recommend that DOACs should be withheld 1 to 2 days before surgical procedures depending on the individual agents and the risk levels of the procedures [13–16, 87]. In the case of an emergency procedure, the risk of bleeding should be weighed against the urgency of intervention [13–16]. After the procedure, DOACs can generally be resumed as soon as adequate hemostasis has been established, and the anticoagulation effect will take place quickly, typically in 1 to 2 hours after restarting the medications [13–16].

Although specific reversal agents for most DOACs are currently lacking, several nonspecific reversal approaches may be applicable to the reversal of DOACs in case of emergency or serious bleeding. Dabigatran, which is 35% protein-bound, may be removed by dialysis as recommended by the prescribing information in the package insert [14, 51]. However, dialysis is ineffective in removing rivaroxaban, apixaban, or edoxaban, which exist in higher protein-bound forms [57, 91–93]. Some evidence suggests that activated charcoal may reduce absorption of dabigatran, rivaroxaban, and apixaban if administered 1 to 2 hours after ingestion [13, 15, 52, 53, 94].

Except for vitamin K, most hemostatic agents used for managing warfarin-related bleeding are applicable to the reversal of DOACs [34, 63, 66, 95–98]. In several human volunteer studies, 4F-PCC has demonstrated effectiveness in reversing the anticoagulant effect of rivaroxaban, apixaban, and edoxaban, as determined by measurements of prothrombin time (PT) or thrombin generation-ETP [63, 66, 95, 97]. The reversal of edoxaban by 4F-PCC has also been established by punch biopsy, an invasive method that may be more representative of clinical bleeding situations than measurements of coagulation parameters [66]. However, 4F-PCC had no effect on dabigatran-induced changes in aPTT, ETP lag time, thrombin clotting time, or ECT [63]. Factor VIII Inhibitor Bypassing Activity (a commercial anti-inhibitor coagulant complex) and an active recombinant form of factor VII have also been tested for feasibility as DOAC reversal agents and have reversed DOAC-induced changes in some coagulation parameters [96–99].

In addition to PCC, tranexamic acid—a synthetic analog of the amino acid lysine, which inhibits fibrinolysis—may be used as an adjuvant therapy to manage bleeding in patients receiving dabigatran [100, 101]. However, this agent is not expected to reverse the anticoagulant activity of edoxaban [16]. Although direct reversal of rivaroxaban by tranexamic acid has not been reported, 1 clinical study suggests that this agent may reduce postoperative blood loss in patients who undergo total hip replacement and receive rivaroxaban for thromboprophylaxis [102]. The effect of tranexamic acid in patients receiving apixaban has not been reported.

The only FDA-approved DOAC-specific reversal agent is Praxbind® (idarucizumab, Boehringer Ingelheim Pharmaceuticals, Inc., Ridgefield, CT), which is indicated to reverse the anticoagulant effects of dabigatran in situations of emergency surgery/urgent procedures or life-threatening/uncontrolled bleeding [35]. Praxbind is a monoclonal antibody fragment that binds dabigatran with an affinity that is 350 times higher than thrombin [103]. Thus, this agent is expected to bind free and thrombin-bound dabigatran and neutralize its activity [103]. In a recent clinical study, idarucizumab reversed the anticoagulant effect of dabigatran to a near complete extent in patients who experienced serious bleeding or required an urgent procedure [103].

**Figure 1:**
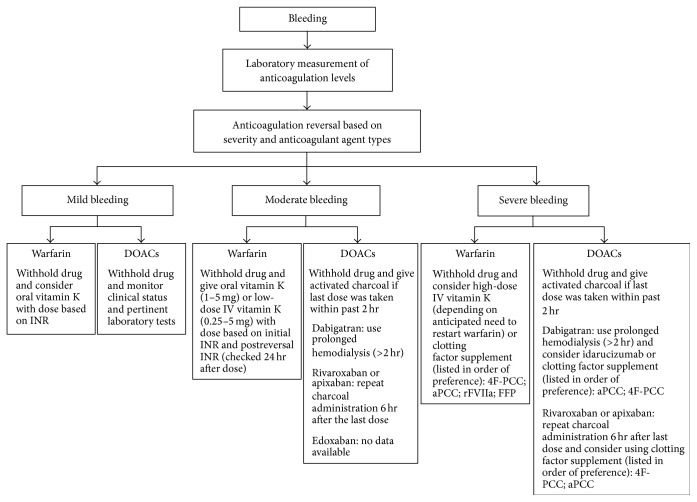
Bleeding management in patients receiving warfarin or DOACs [10, 34–38]. 4F-PCC, 4-factor prothrombin complex concentrate; aPCC, activated prothrombin complex concentrate; DOACs, direct-acting oral anticoagulants; FFP, fresh frozen plasma; INR, international normalized ratio; IV, intravenous; rFVIIa, recombinant activated factor VII.

Several DOAC-specific antidotes are under development and have shown promise in preliminary studies. PER977 (Perosphere, Inc., Danbury, CT) [104, 105], a small synthetic molecule with binding activity to all 4 DOACs, reduces the whole-blood clotting time prolonged by edoxaban [106]. Further, andexanet alfa (PRT064445, Portola Pharmaceuticals, Inc., South San Francisco, CA), a recombinant catalytically inactive FXa decoy molecule, may provide a rapid and sustained reversal of DOACs as measured by anti-Xa activity in subjects taking apixaban and rivaroxaban [105, 107–110]. Ongoing clinical studies are evaluating the effect of andexanet alfa on edoxaban [105]. Bleeding management in patients receiving warfarin or DOACs is illustrated in Figure 1.

Thromboembolic complications are the primary concern when anticoagulant reversal agents are used. Infusion of PCC may lead to the development of thrombotic stroke, DVT, and myocardial infarction [111, 112], although a patient's underlying thrombotic risk factors may also contribute to these AEs [111]. Thrombotic events such as DVT, PE, myocardial infarction, and ischemic stroke have also been reported in patients receiving idarucizumab [103]. The safety of PER977 and andexanet alfa is still under evaluation in several ongoing clinical studies [105]. No evidence has shown that the use of reversal agents reduces patient mortality.

## 6. Implications for Care

### 6.1. DOAC Cost-Effectiveness

The costs of DOACs average from $300 to $600 per month depending on the specific DOAC type and the dosing regimens [113]. However, there are also high costs associated with NVAF-related stroke and VTE. The estimated 1-time cost of ischemic stroke in AF is $15,497 with moderate-to-severe sequelae and $9,562 with minor sequelae. Furthermore, the continuing monthly costs of AF-related ischemic stroke with moderate-to-severe sequelae and minor sequelae are $5,605 and $2,528, respectively (costs adjusted from 2008 US dollars to 2011 US dollars) [113]. The costs for a single event of DVT and PE are $7,712 and $9,566, respectively, accounting for the expense of both immediate and follow-up treatment for VTE [4, 114].

Cost-effectiveness analyses of DOACs versus warfarin have been performed [115, 116]. For stroke prevention in NVAF, the annual total cost reduction associated with the use of dabigatran, rivaroxaban, apixaban, and edoxaban relative to warfarin is $204, $140, $495, and $340 per patient, respectively [115]. This overall reduction is primarily attributed to the decreased incidence and incremental costs associated with major bleeding [115]. In a hypothetical insured population of 1 million people, including both NVAF and VTE patients, the medical cost reductions resulting from DOACs relative to warfarin are estimated to be $3.0, $2.1, $7.3, and $5.0 million for NVAF patients treated with dabigatran, rivaroxaban, apixaban, and edoxaban, respectively; $0.7, $2.2, $4.1, and $1.6 million for patients with acute VTE treated with dabigatran, rivaroxaban, apixaban, and edoxaban, respectively; and $3.7, $4.2, $11.5, and $6.6 million for the whole population treated with dabigatran, rivaroxaban, apixaban, and edoxaban, respectively [115]. The medical cost reductions associated with DOAC use are projected to steadily increase [115]. On the other hand, cost-effectiveness analyses are predominantly based on data from the phase 3 clinical trials and may not represent the use of DOACs in a real-world setting [115]. With the increasing adoption of DOACs, data derived from routine clinical practice will bring additional insights into the cost considerations for choice of oral anticoagulant use [117].

### 6.2. Outpatient Management of VTE Using DOACs

The use of DOACs facilitates the transition of VTE management from hospitals or emergency departments to a patient's home. The ACCP guidelines recommend initial home treatment for patients with lower limb DVT and early discharge for patients with low-risk PE if the patient's home circumstances are adequate [6]. The required circumstances include well-maintained living conditions, strong support from family or friends, phone access, and the ability to quickly return to the hospital if deterioration occurs [6]. Home care is associated with the benefits of comfort, convenience, and significant cost savings [6]. Outpatient management of VTE is also supported by clinical evidence. A study comparing home versus in-hospital initial treatment of outpatients with acute DVT revealed that home treatment was associated with a lower rate of major bleeding or death than in-hospital treatment [118], although this conclusion may be confounded by the fact that the home-treated patients in this study were younger and had fewer comorbidities than the hospital-treated ones [118]. Additionally, a US and European open-label, randomized noninferiority trial indicated that for selected low-risk patients with PE, outpatient treatment could be as effective and safe as inpatient treatment [119]. A recently published study conducted in 2 US emergency departments has established the effectiveness of DOACs in VTE outpatient treatment. In this study, patients with low-risk DVT or PE were discharged immediately after diagnosis, received home treatment with rivaroxaban, and demonstrated a low rate of VTE recurrence and bleeding [120].

Patients with VTE suffer from impaired quality of life (QOL) [121]. A study conducted in patients with newly diagnosed VTE or AF discovered that patients' QOL was significantly improved at 3 months after the initiation of VKA therapy [122]. While the study did not address whether the initial low QOL was due to the disease diagnosis or the start of VKA treatment, the investigators speculated that the improvement in QOL may result from “the patient's acceptance of the diagnosis as well as the benefits of treatment” [122]. The impact of DOACs on patients' QOL is not yet well studied. Limited evidence suggests that dabigatran and warfarin have similar impacts on QOL, which was unexpected by the study investigators because of “the known complexities of warfarin treatment” [123]. As DOACs are easy to use, with fewer food and drug interactions and reduced monitoring requirements compared with warfarin, they are expected to improve patients' QOL. Additional research is necessary to provide a better understanding of the impact of DOACs on patients' QOL.

## 7. Conclusions

Four DOACs are available for the prevention of stroke in patients with NVAF and for the treatment of VTE. Compared with the traditional oral anticoagulant warfarin, advantages of the DOACs include faster onset of action, fewer food and drug interactions, and lower requirements for monitoring. Clinical studies have demonstrated that DOACs are at least as effective as warfarin for the prevention of NVAF-related stroke, the treatment of acute VTE, and the prevention of recurrent VTE and are associated with similar or decreased risks of bleeding. Despite these advantages, several important factors should be taken into consideration regarding the appropriate use of DOACs in clinical practice. Different DOACs vary in dose regimens, and dose adjustments are necessary in patients with low body weight, renal impairment, or concomitant medications. Bleeding is a concern for all anticoagulants, and the lack of specific laboratory measurements and reversal agents poses additional challenges to its management in patients using DOACs. If bleeding occurs, knowledge of nonspecific measurements and reversal approaches may mitigate AEs associated with DOACs. Overall, DOACs represent effective, safe, and likely cost-effective alternatives to warfarin. Appropriate use of DOACs will maximize their benefits and improve the prevention of NVAF-related stroke and the treatment of VTE.

## Supplementary Material

The primary efficacy and safety endpoints of the pivotal phase 3 clinical trials in patients with nonvalvular atrial fibrillation (NVAF) demonstrated that direct-acting oral anticoagulants (DOACs) were at least as effective as warfarin in reducing the risk of stroke or systemic embolic events and were associated with similar or significantly decreased rates of bleeding (Supplementary Table 1). The primary efficacy and safety endpoints of the pivotal phase 3 clinical trials in patients with venous thromboembolism (VTE) demonstrated that DOACs were noninferior for the treatment of acute symptomatic VTE and were associated with similar or significantly decreased bleeding risks compared with standard therapy (low molecular weight heparin or unfractionated heparin followed by treatment with an overlapping VKA (vitamin K antagonist: warfarin or acenocoumarol)). In addition, VTE patients treated with DOACs and with standard therapy (heparin/VKA) experienced similar recurrence risks (Supplementary Table 2).

## Figures and Tables

**Table 1:** Properties of DOACs [13–16, 28].

	Dabigatran	Rivaroxaban	Apixaban	Edoxaban
Class	Oral thrombin inhibitor	Oral factor Xa inhibitor	Oral factor Xa inhibitor	Oral factor Xa inhibitor

FDA-approved indication	Reduction of stroke and SEE risk for patients with NVAF; treatment of DVT and PE following 5–10 days of parenteral anticoagulant; and reduction of recurrence risk for DVT and PE	Reduction of stroke and SEE risk for patients with NVAF; treatment of DVT and PE and reduction of recurrence risk for DVT and PE; prophylaxis of DVT in patients undergoing knee or hip replacement surgery	Reduction of stroke and SEE risk for patients with NVAF; treatment of DVT and PE and reduction of recurrence risk for DVT and PE; prophylaxis of DVT in patients undergoing knee or hip replacement surgery	Reduction of stroke and SEE risk for patients with NVAF and treatment of DVT and PE following 5–10 days of parenteral anticoagulant^a^

Time to *C*_max_ (h)	1-2	2–4	3-4	1-2

Half-life (h)	12–17	5–13	12	10–14

Renal elimination	80% of absorbed dose	66% of oral dose	27% of absorbed dose	50% of absorbed dose

Transporters^b^	P-gp	P-gp/BCRP	P-gp/BCRP	P-gp

Cytochrome P450 metabolism	No	Yes	Yes	Minimal

Bioavailability (%)	3–7	≥80^c^	50^d^	62

Potential drug interactions	Potent P-gp inhibitors and P-gp/CYP3A4 dual inducer rifampin	Potent dual CYP3A4 and P-gp inhibitors or inducers	Potent dual CYP3A4 and P-gp inhibitors or inducers	Potent P-gp inhibitors and P-gp/CYP3A4 dual inducer rifampin

^a^Edoxaban should not be used in NVAF patients with creatinine clearance >95 mL/min [16].

^b^DOACs are substrates of these transporters.

^c^For 10 mg dose. For 20 mg dose in the fasted state, it is 66%.

^d^For doses up to 10 mg.

BCRP, breast cancer resistance protein; *C*_max_, maximum observed plasma concentration; CYP3A4, cytochrome P450 3A4 enzyme; DOACs, direct-acting oral anticoagulants; DVT, deep-vein thrombosis; NVAF, nonvalvular atrial fibrillation; P-gp, P-glycoprotein; PE, pulmonary embolism; SEE, systemic embolic event; VTE, venous thromboembolism.

**Table 2:** Summary of bleeding outcomes of DOACs from phase 3 clinical trials for the prevention of stroke and SEE in patients with NVAF [19–22].

	Major bleeding		Intracranial bleeding		Gastrointestinal bleeding	
	*N*	HR (95% CI)	*N*	HR (95% CI)	*N*	HR (95% CI)
	(%)	*P* value	(%)	*P* value	(%)	*P* value
RE-LY						
Dabigatran 150 mg BID	375	0.93 (0.81–1.07)	36	0.40 (0.27–0.60)	182	1.50 (1.19–1.89)
(*n* = 6076)	(3.11)	0.31	(0.30)	<0.001	(1.51)	<0.001
Dabigatran 110 mg BID	322	0.80 (0.69–0.93)	27	0.31 (0.20–0.47)	133	1.10 (0.86–1.41)
(*n* = 6015)	(2.71)	0.003	(0.23)	<0.001	(1.12)	0.43
Warfarin	397		87		120	
(*n* = 6022)	(3.36)		(0.74)		(1.02)	
ROCKET AF						
Rivaroxaban 20 mg QD^a^	395	1.04 (0.90–1.20)	55	0.67 (0.47–0.93)	224	NR
(*n* = 7111)	(5.60)	0.58	(0.80)	0.02	(3.15)	
Warfarin	386		84		154	
(*n* = 7125)	(5.40)		(1.20)		(2.16)	
ARISTOTLE						
Apixaban 5 mg BID^b^	327	0.69 (0.60–0.80)	52	0.42 (0.30–0.58)	105	0.89 (0.70–1.15)
(*n* = 9088)	(2.13)	<0.001	(0.33)	<0.001	(0.76)	0.37
Warfarin	462		122		119	
(*n* = 9052)	(3.09)		(0.80)		(0.86)	
ENGAGE AF-TIMI 48						
Edoxaban 60 mg QD	418	0.80 (0.71–0.91)	61	0.47 (0.34–0.63)	232	1.23 (1.02–1.50)
(*n* = 7012)	(2.75)	<0.001	(0.39)	<0.001	(1.51)	0.03
Edoxaban 30 mg QD	254	0.47 (0.41–0.55)	41	0.30 (0.21–0.43)	129	0.67 (0.53–0.83)
(*n* = 7002)	(1.61)	<0.001	(0.26)	<0.001	(0.82)	<0.001
Warfarin	524		132		190	
(*n* = 7012)	(3.43)		(0.85)		(1.23)	

^a^15 mg QD in patients with creatinine clearance 30–49 mL/min.

^b^2.5 mg BID in patients meeting 2 or more of the following criteria: age ≥80 years, body weight ≤60 kg, or serum creatinine ≥15 mg/L.

ARISTOTLE, apixaban for reduction in stroke and other thromboembolic events in atrial fibrillation; BID, twice daily; CI, confidence interval; DOACs, direct-acting oral anticoagulants; ENGAGE AF-TIMI 48, effective anticoagulation with factor Xa next generation in atrial fibrillation-thrombolysis in myocardial infarction 48; HR, hazard ratio; NR, not reported; QD, once daily; NVAF, nonvalvular atrial fibrillation; RE-LY, randomized evaluation of long-term anticoagulation therapy; ROCKET AF, rivaroxaban once daily oral direct factor Xa inhibition compared with vitamin K antagonism for prevention of stroke and embolism trial in atrial fibrillation; SEE, systemic embolic event.

**Table 3:** Summary of bleeding outcomes of DOACs from phase 3 clinical trials for the treatment and secondary prevention of VTE [29–33].

	Major bleeding		Clinically relevant bleeding	
	*N* (%)	HR (95% CI) *P* value	*N*(%)	HR (95% CI) *P* value
RECOVER I/II				
Dabigatran 150 mg BID^a^	37	0.73 (0.48–1.11)	136	0.62 (0.50–0.76)
(*n* = 2553)	(1.4)	NR	(5.3)	NR
Heparin/VKA	51		217	
(*n* = 2554)	(2.0)		(8.5)	
EINSTEIN-DVT				
Rivaroxaban^b^	14	0.65 (0.33–1.30)	139	0.97 (0.76–1.22)
(*n* = 1718)	(0.8)	0.21	(8.1)	0.77
Heparin/VKA	20		138	
(*n* = 1711)	(1.2)		(8.1)	
EINSTEIN-PE				
Rivaroxaban^b^	26	0.49 (0.31–0.79)	249	0.90 (0.76–1.07)
(*n* = 2412)	(1.1)	0.003	(10.3)	0.23
Heparin/VKA	52		274	
(*n* = 2405)	(2.2)		(11.4)	
AMPLIFY				
Apixaban^c^	15	0.31 (0.17–0.55)	115	0.44 (0.36–0.55)
(*n* = 2676)	(0.6)	<0.001	(4.3)	<0.001
Heparin/VKA	49		261	
(*n* = 2689)	(1.8)		(9.7)	
Hokusai-VTE				
Edoxaban 60 mg QD^a,d^	56	0.84 (0.59–1.21)	349	0.81 (0.71–0.94)
(*n* = 4118)	(1.4)	0.35	(8.5)	0.004
Heparin/VKA	66		423	
(*n* = 4122)	(1.6)		(10.3)	

^a^With a parenteral anticoagulation lead-in.

^b^15 mg BID for 3 weeks followed by 20 mg QD.

^c^10 mg BID for the first 7 days followed by 5 mg BID for 6 months.

^d^30 mg QD in patients with creatinine clearance 30–50 mL/min or body weight ≤60 kg or receiving concomitant potent P-glycoprotein inhibitors.

AMPLIFY, apixaban for the initial management of pulmonary embolism and deep-vein thrombosis as first-line therapy; BID, twice daily; CI, confidence interval; DOACs, direct-acting oral anticoagulants; DVT, deep-vein thrombosis; HR, hazard ratio; NR, not reported; PE, pulmonary embolism; QD, once daily; VKA, vitamin K antagonist; VTE, venous thromboembolism.

**Table 4:** DOAC dosing for treatment and secondary prevention of VTE in the US [13–16].

Patient population	Dabigatran	Rivaroxaban	Apixaban	Edoxaban
General population	150 mg twice daily after 5–10 days of initial parenteral therapy if CrCl >30 mL/min	15 mg^a^ twice daily for 21 days and then 20 mg^a^ once daily	10 mg twice daily for 7 days and then 5 mg twice daily	60 mg once daily following 5–10 days of initial parenteral anticoagulant therapy

Renal impairment	No recommendations if CrCl ≤30 mL/min or on dialysis	Avoid if CrCl <30 mL/min	No dose change	Reduce dose to 30 mg once daily if CrCl is 15–50 mL/min

Elderly	No dose change^b^	No dose change^b^	No dose change	No dose change

Low body weight	NR	No dose change	NR	Reduce dose to 30 mg once daily if weight ≤60 kg

Concomitant P-gp inhibitor	Avoid if CrCl <50 mL/min	Avoid if P-gp inhibitor is also a strong CYP3A4 inhibitor	Reduce to 5.0 or 2.5 mg (for 10.0 and 5.0 mg doses, resp.) if P-gp inhibitor is also a strong CYP3A4 inhibitor; avoid if already taking 2.5-mg dose	Reduce dose to 30 mg once daily

Concomitant P-gp inducer	Avoid (e.g., rifampin)	Avoid if P-gp inducer is also a strong CYP3A4 inducer	Avoid if P-gp inducer is also a strong CYP3A4 inducer	Avoid concomitant use with rifampin

^a^Should be taken with food.

^b^Risk of stroke and bleeding increases with age but risk/benefit is favorable.

CrCl, creatinine clearance; CYP3A4, cytochrome P450 3A4 enzyme; DOAC, direct-acting oral anticoagulant; NR, not reported; P-gp, P-glycoprotein; VTE, venous thromboembolism.
