# Supplementary material for: Direct-Acting Oral Anticoagulants: Practical Considerations for Emergency Medicine Physicians
Source: Emerg Med Int. 2016 May 16;2016:1781684. doi: 10.1155/2016/1781684 (PMC4884797; doi:10.1155/2016/1781684)
Supplement: Supplementary file 1 — The primary efficacy and safety endpoints of the pivotal phase 3 clinical trials in patients with nonvalvular atrial fibrillation (NVAF) demonstrated that direct-acting oral anticoagulants (DOACs) were at least as effective as warfarin in reducing the risk of stroke or systemic embolic events and were associated with similar or significantly decreased rates of bleeding (Supplementary Table 1). The primary efficacy and safety endpoints of the pivotal phase 3 clinical trials in patients with venous thromboembolism (VTE) demonstrated that DOACs were noninferior for the treatment of acute symptomatic VTE and were associated with similar or significantly decreased bleeding risks compared with standard therapy (low molecular weight heparin or unfractionated heparin followed by treatment with an overlapping VKA (vitamin K antagonist: warfarin or acenocoumarol)). In addition, VTE patients treated with DOACs and with standard therapy (heparin/VKA) experienced similar recurrence risks (Supplementary Table 2). [file 1781684.f1.pdf]

Supplemental Table 1: Summary of safety and efficacy of DOACs for the prevention of stroke and SEE in patients with NVAF [19-22]

	Primary efficacy endpoint[a]		Primary safety outcome[b]	
	N, n (%)	HR (95% CI) *P* value	N, n (%)	HR (95% CI) *P* value
RE-LY				
Dabigatran 110 mg BID	6015, 182 (1.53)	0.91 (0.74–1.11) 0.34	6015, 322 (2.71)	0.80 (0.69–0.93) 0.003
Dabigatran 150 mg BID	6076, 134 (1.11)	0.66 (0.53–0.82) <0.001	6076, 375 (3.11)	0.93 (0.81–1.07) 0.31
Warfarin	6022, 199 (1.69)		6022, 397 (3.36)	
ROCKET AF				
Rivaroxaban 20 mg QD[c]	7081, 269 (2.10)	0.88 (0.75–1.03) 0.12	7111, 1475 (20.70)	1.03 (0.96–1.11) 0.44
Warfarin	7090, 306 (2.40)		7125, 1449 (20.30)	
ARISTOTLE				
Apixaban 5 mg BID[d]	9120, 212 (1.27)	0.79 (0.66–0.95) 0.01	9088, 327 (2.13)	0.69 (0.60–0.80) <0.001
Warfarin	9081, 265 (1.60)		9052, 462 (3.09)	
ENGAGE AF-TIMI 48				
Edoxaban 60 mg QD	7035, 182 (1.18)	0.79 (0.63–0.99)[e] <0.001[f]	7012, 418 (2.75)	0.80 (0.71–0.91) <0.001
Edoxaban 30 mg QD	7034, 253 (1.61)	1.07 (0.87–1.31)[e] 0.005[f]	7002, 254 (1.61)	0.47 (0.41–0.55) <0.001
Warfarin	7036, 232 (1.50)		7012, 524 (3.43)	

[a]For RE-LY, ROCKET AF, ARISTOTLE, and ENGAGE AF-TIMI 48, the primary efficacy endpoint was stroke or SEE.

[b]For RE-LY, ARISTOTLE, and ENGAGE AF-TIMI 48, the primary safety endpoint was major bleeding; for ROCKET AF, the primary safety endpoint was major or clinically relevant nonmajor bleeding.

[c]15 mg QD in patients with creatinine clearance 30–49 mL/min.

[d]2.5 mg BID in patients meeting 2 or more of the following criteria: age ≥80 years, body weight ≤60 kg, or serum creatinine ≥15 mg/L.

[e]97.5% CI.

[f]P value for noninferiority.

ARISTOTLE, Apixaban for Reduction in Stroke and Other Thromboembolic Events in Atrial Fibrillation; BID, twice daily; DOACs, direct-acting oral anticoagulants; ENGAGE AF-TIMI 48, Effective Anticoagulation with Factor Xa Next Generation in Atrial Fibrillation–Thrombolysis in Myocardial Infarction 48; HR, hazard ratio; N, number of total patients; n, number of events;

NVAF, nonvalvular atrial fibrillation; QD, once daily; RE-LY, Randomized Evaluation of Long-Term Anticoagulation Therapy; ROCKET AF, Rivaroxaban Once Daily Oral Direct Factor Xa Inhibition Compared with Vitamin K Antagonism for Prevention of Stroke and Embolism Trial in Atrial Fibrillation; SEE, systemic embolic event; VKA, vitamin K antagonist.

Supplemental Table 2: Summary of safety and efficacy of DOACs for the treatment and secondary prevention of VTE [28-32]

	Primary efficacy endpoint[a]		Primary safety outcome[b]	
	N, n (%)	HR (95% CI) *P* value[c]	N, n (%)	HR (95% CI) *P* value
RE-COVER I/II				
Dabigatran 150 mg BID[d]	2553, 60 (2.4)	1.09 (0.76–1.57) NR	2553, 37 (1.4)	0.73 (0.48–1.11) NR
Heparin/VKA	2554, 55 (2.2)		2554,51 (2.0)	
EINSTEIN-DVT				
Rivaroxaban[e]	1731, 36 (2.1)	0.68 (0.44–1.04) <0.001	1718, 139 (8.1)	0.97 (0.76–1.22) 0.77
Heparin/VKA	1718 51 (3.0)		1711 138 (8.1)	
EINSTEIN-PE				
Rivaroxaban[e]	2419, 50 (2.1)	1.12 (0.75–1.68) 0.003	2412, 249 (10.3)	0.90 (0.76–1.07) 0.23
Heparin/VKA	2413 44 (1.8)		2405 274 (11.4)	
AMPLIFY				
Apixaban[f]	2609, 59 (2.3)	0.84 (0.60–1.18) <0.001	2676, 15 (0.6)	0.31 (0.17–0.55) <0.001
Heparin/VKA	2635 71 (2.7)		2689, 49 (1.8)	
Hokusai-VTE				
Edoxaban 60 mg QD[d,g]	4118, 130 (3.2)	0.89 (0.70–1.13) <0.001	4118, 349 (8.5)	0.81 (0.71–0.94) 0.004
Heparin/VKA	4122, 146 (3.5)		4122, 423 (10.3)	

[a]For Hokusai-VTE, AMPLIFY, and RE-COVER I/II, the primary efficacy endpoint was first recurrent VTE or VTE-related death; for EINSTEIN-DVT and EINSTEIN-PE, the primary efficacy endpoint was recurrent VTE.

[b]For Hokusai-VTE, EINSTEIN-PE, and EINSTEIN-DVT, the primary safety endpoint was major or clinically relevant nonmajor bleeding; for AMPLIFY and RECOVERI/II, the primary safety endpoint was major bleeding.

[c]P value for noninferiority.

[d]With a parenteral anticoagulation lead-in.

[e]15 mg BID for 3 weeks followed by 20 mg QD.

[f]10 mg BID for the first 7 days followed by 5 mg BID for 6 months.

[g]30 mg QD in patients with creatinine clearance 30–50 mL/min or body weight ≤60 kg, or receiving concomitant potent P-glycoprotein inhibitors.

AMPLIFY, Apixaban for the Initial Management of Pulmonary Embolism and Deep-Vein Thrombosis as First-Line Therapy; BID, twice daily; CI, confidence interval; DOACs, direct-

acting oral anticoagulants; DVT, deep vein thrombosis; HR, hazard ratio; N, number of total patients; n, number of events; NR, not reported; PE, pulmonary embolism; QD, once daily; VKA, vitamin K antagonist; VTE, venous thromboembolism.
